# Supplementary material for: Angle-resolved photoemission spectroscopy of superconducting (La,Pr)3Ni2O7/SrLaAlO4 heterostructures
Source: Natl Sci Rev. 2025 May 21;12(10):nwaf205. doi: 10.1093/nsr/nwaf205 (PMC12485603; doi:10.1093/nsr/nwaf205)
Supplement: nwaf205_Supplemental_File [file nwaf205_supplemental_file.docx]

Supplementary Materials for

**Angle-resolved photoemission spectroscopy of superconducting (La_,_Pr)_3_Ni_2_O_7_/SrLaAlO_4_ heterostructures**

Peng Li^1,2†^, Guangdi Zhou^1,2†^, Wei Lv^2†^, Yueying Li^2†^, Changming Yue^1,2,3*^, Haoliang Huang^1,2^, Lizhi Xu^2^, Jianchang Shen^4,5^, Yu Miao^4,5^, Wenhua Song^2^, Zihao Nie^2^, Yaqi Chen^2^, Heng Wang^2^, Weiqiang Chen^1,2^, Yaobo Huang^6^, Zhen-Hua Chen^6^, Tian Qian^7^, Junhao Lin^1,2^, Junfeng He^4,5*^, Yu-Jie Sun^1,2*^, Zhuoyu Chen^1,2*^, Qi-Kun Xue^1,2,8^

^†^These authors contributed equally.

^*^E-mail: chenzhuoyu@sustech.edu.cn, sunyj@sustech.edu.cn, jfhe@ustc.edu.cn, yuecm@sustech.edu.cn


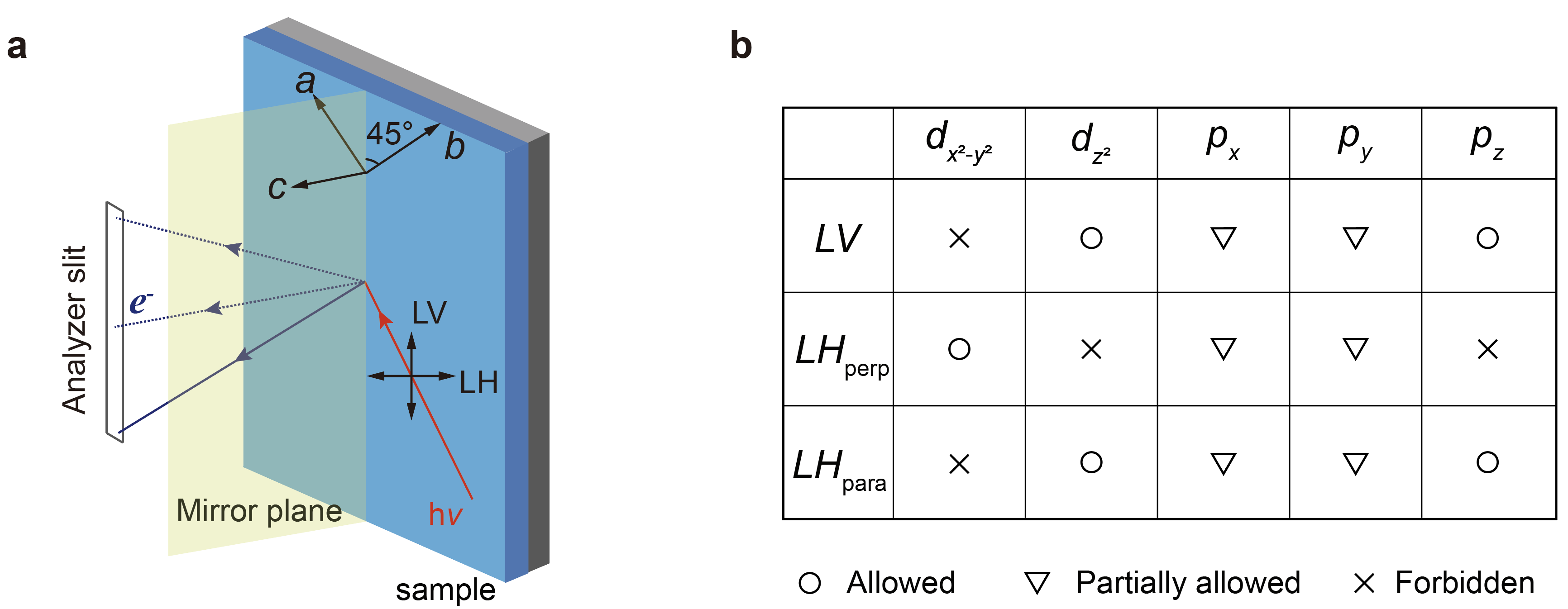


**Figure S1** **Matrix element effect.** **a**, The geometry of the ARPES measurements with polarized photons at the beamline. The mirror plane is parallel to the (11) direction of the sample surface. **b**, The possibility to detect various Ni-*d* and O-*p* orbitals at the mirror plane with LV and LH photons, based on matrix element analysis. The LV polarization direction is always parallel to the mirror plane. For LH polarized photons, there are two components, one perpendicular (denoted “perp”) to the mirror plane and the other one parallel (denoted “para”) to the mirror plane. The ARPES results from LH photons is the summation of both components, thus allowing more orbitals to be observed.


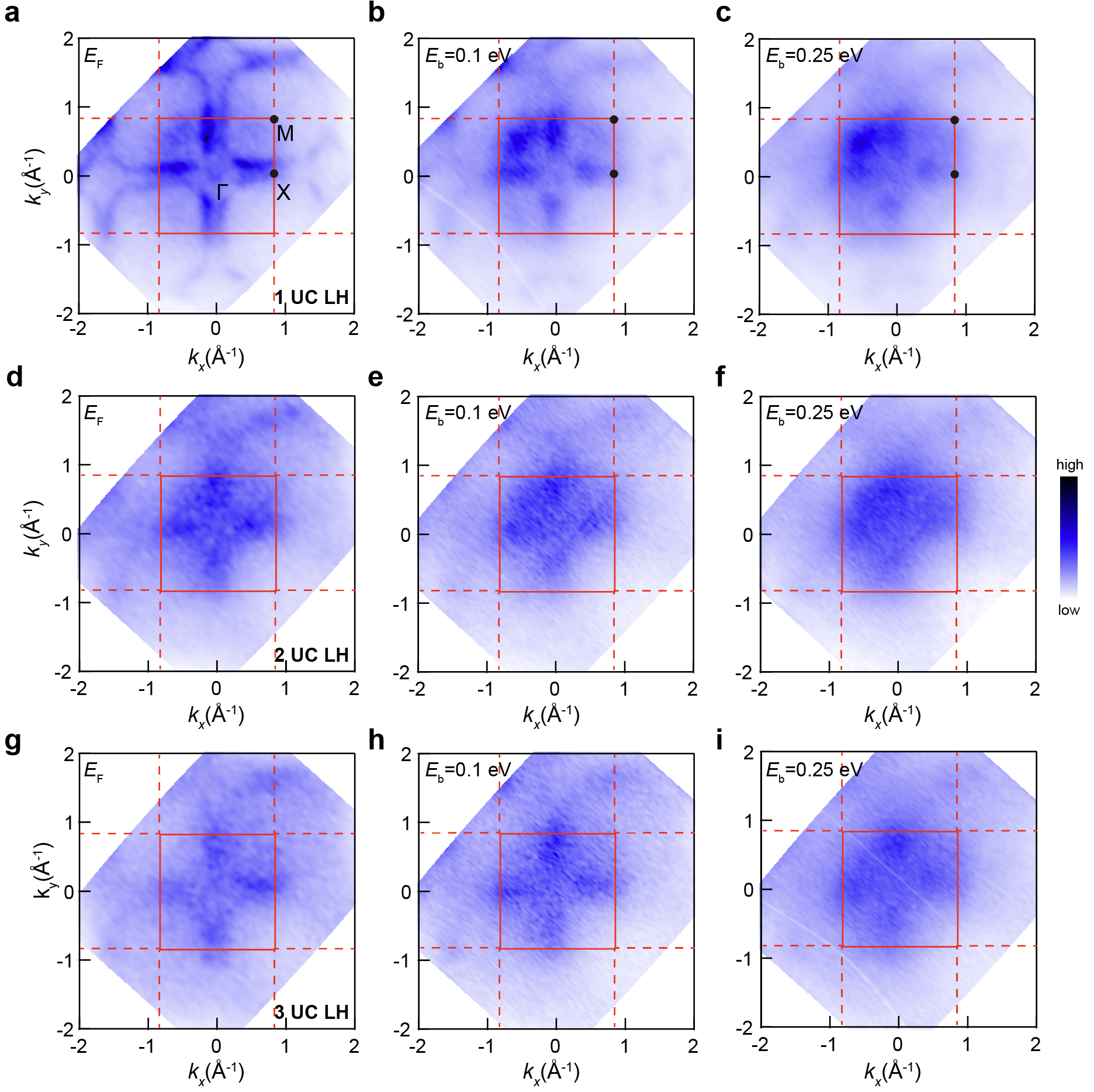


**Figure S2 LH constant energy contours.** Intensity integrated within ±50 meV energy window at specific binding energies with LH polarized photons of 1UC (**a-c**), 2UC (**d-f**) and 3UC (**g-i**) heterostructures. The consistent constant energy contours across 1UC, 2UC, and 3UC further confirm comparable carrier doping level in all three films.


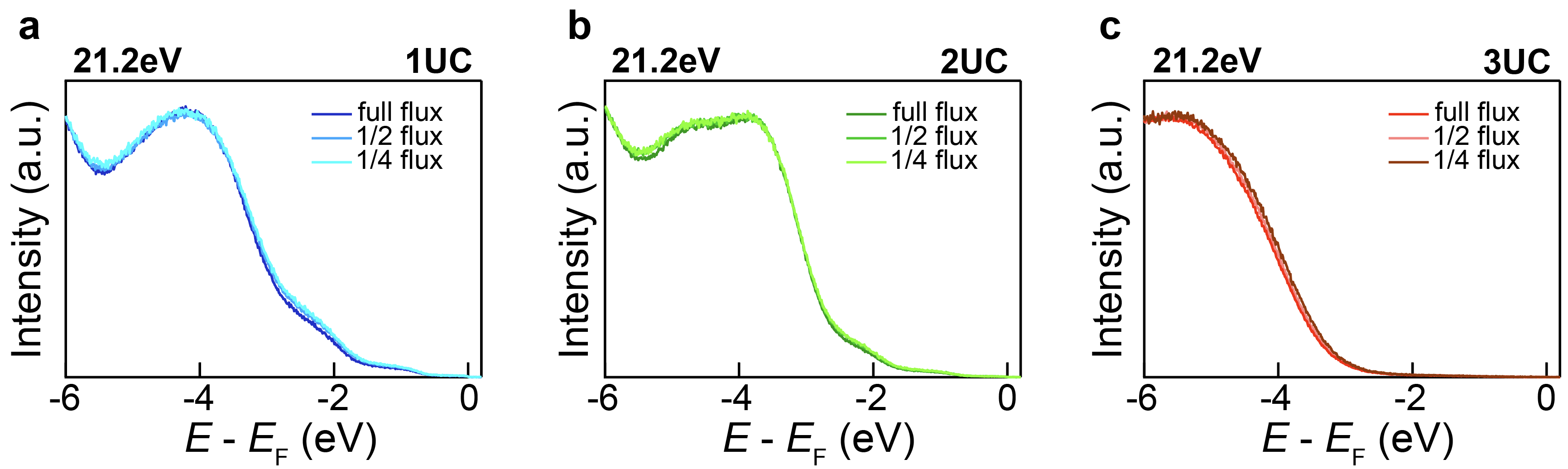


**Figure S3 Flux dependence.** Flux-dependent momentum-integrated energy distribution curves (EDCs) of 1UC (**a**), 2UC (**b**), and 3UC (**c**) samples using 21.2 eV photons. The 1/4, 1/2 and full flux indicate different ​amounts of injected photons per unit time. If the samples are completely insulating with charging accumulation, the variance of photon flux would lead to different photoelectron intensities and peak positions, which is not the case shown here, implying the existence of conductive channels.


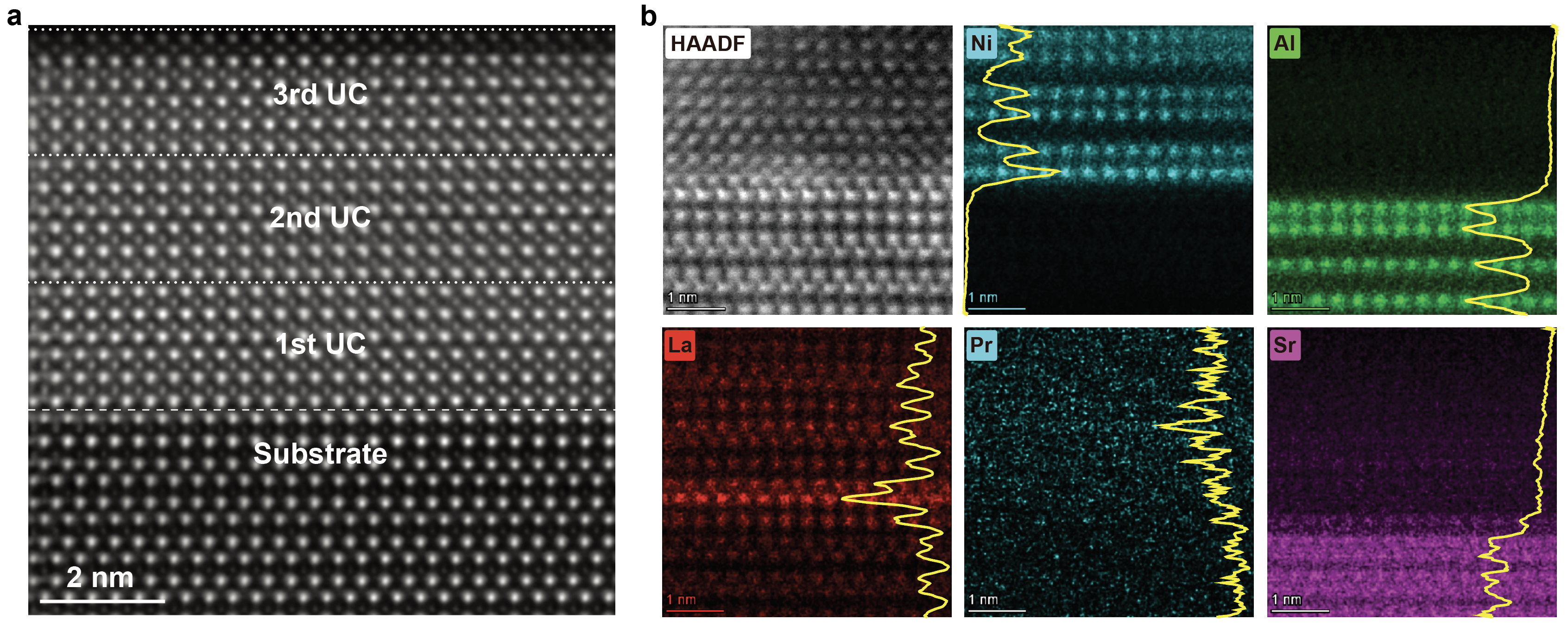


**Figure S4 STEM and EDS.** Same STEM HAADF (**a**) and atomically resolved EDS (**b**) images shown in Fig. 2 in the main text, with larger field of view and added La, Pr data.


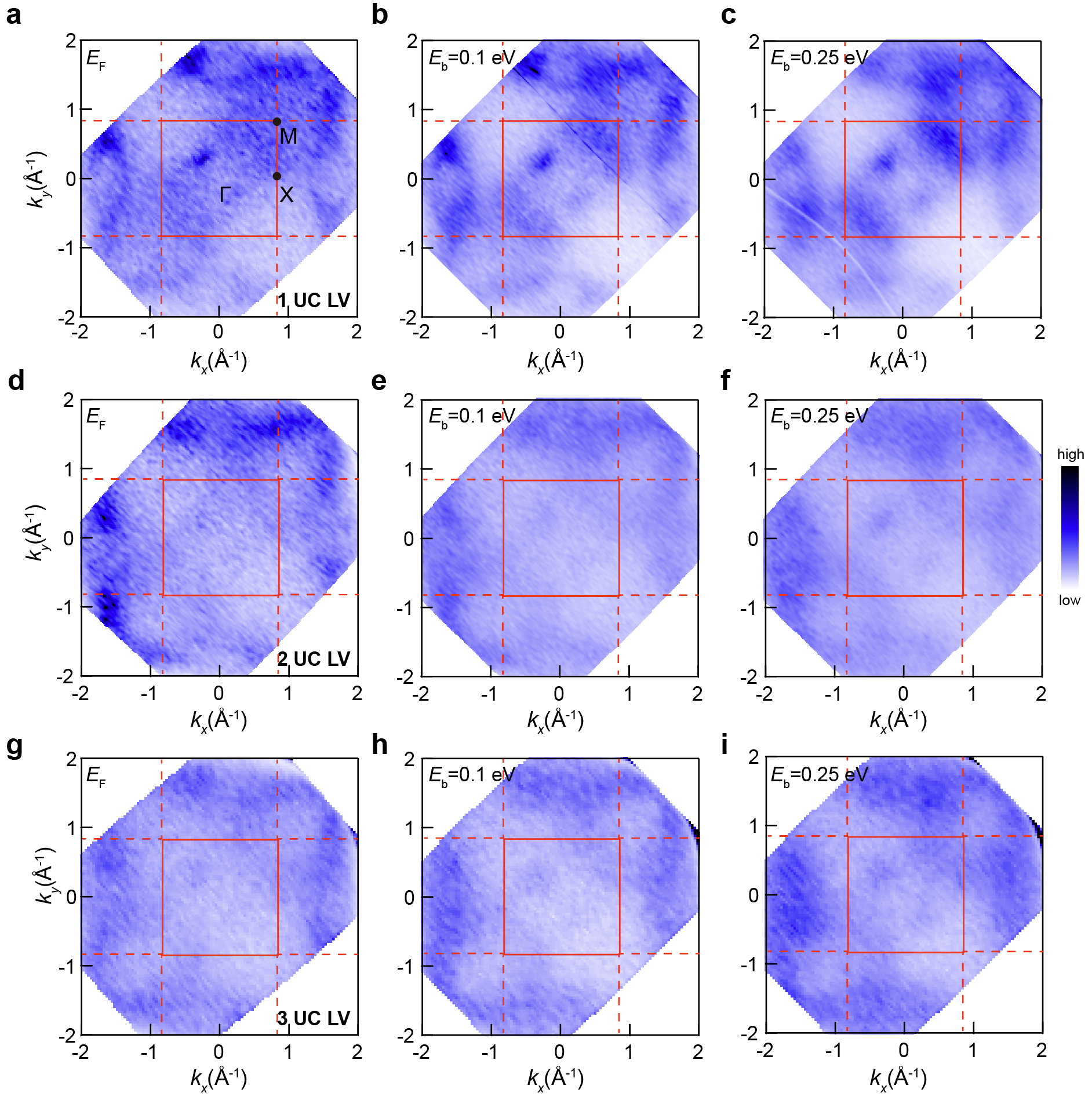


**Figure S5** **LV constant energy contours.** Intensity integrated within ±50 meV energy window at specific binding energies with LV polarized photons of 1UC (**a-c**), 2UC (**d-f**) and 3UC (**g-i**) heterostructures. The consistent constant energy contours across 1UC, 2UC, and 3UC further confirm comparable carrier doping level in all three films.


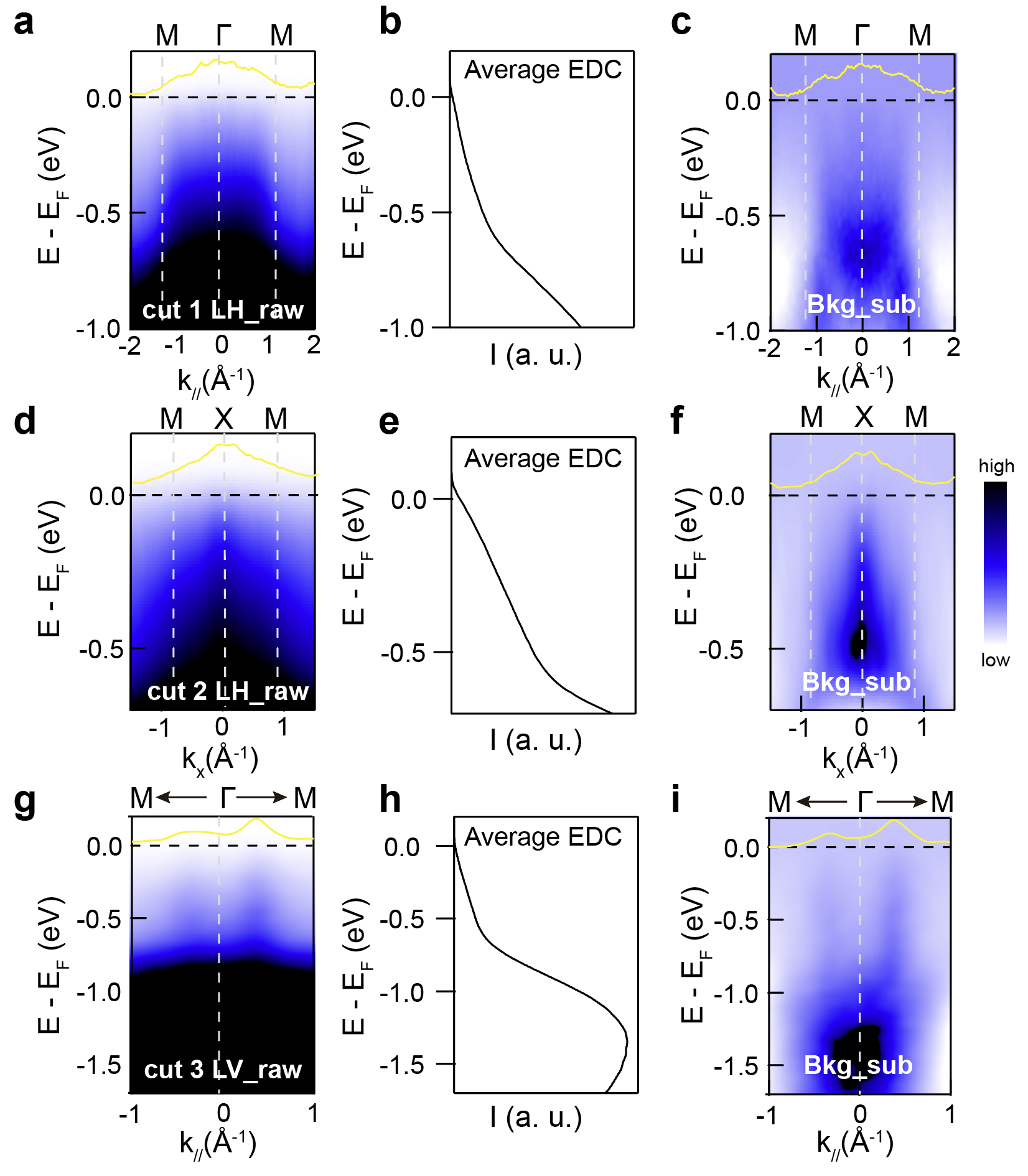


**Figure S6** **Background substraction.** **a,** **d, g,** Raw data of cuts 1, 2, and 3 in Fig. 3, respectively, with yellow curves representing the raw MDCs at the Fermi level. **b, e, h,** The corresponding average EDCs as the background. **c, f, i,** Spectra after substracting the background to enhance features, same data as the Figs. 3g, 3h and 3i.


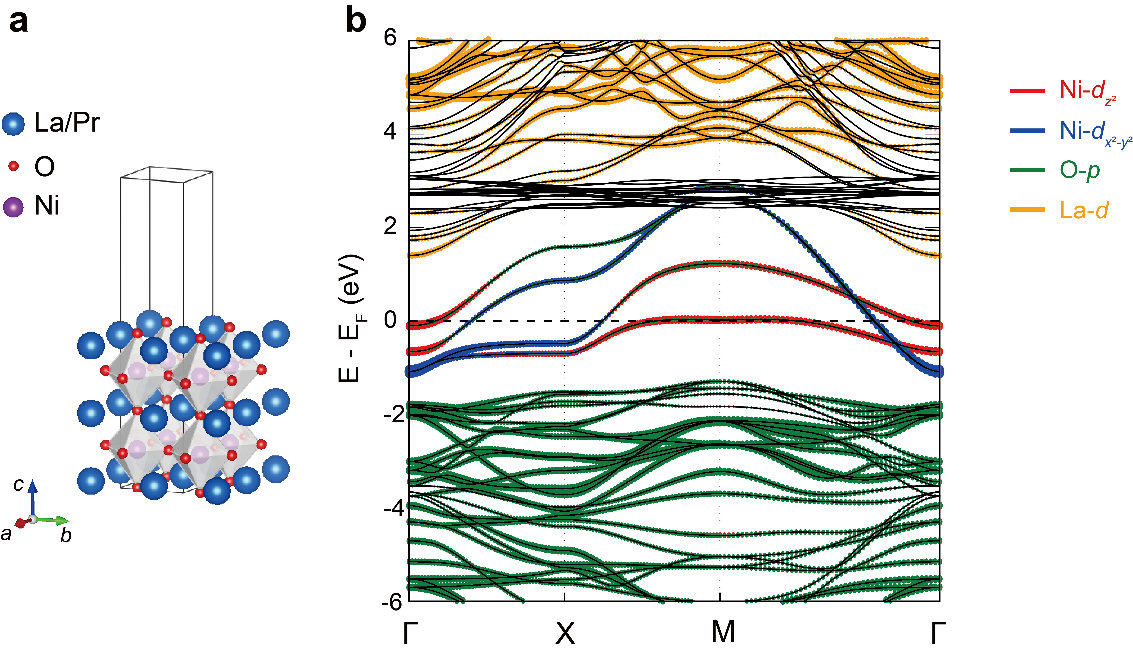


**Figure S7 DFT+U results with orbital characters.** **a,** The crystal structure of one-bilayer-thick La_3_Ni_2_O_7_ thin film constructed with experimentally determined lattice constants [42]. The actual length of vacuum in the calculation is more than 30 Angstrom (much bigger than drawn). **b,** The DFT band structure characterized by different orbitals.
